# Towards a computational model of Dyslexia

**DOI:** 10.1186/1471-2202-16-S1-O12

**Published:** 2015-12-18

**Authors:** Sagi Jaffe-Dax, Ofri Raviv, Nori Jacoby, Yonatan Loewenstein, Merav Ahissar

**Affiliations:** 1 Edmond and Lily Safra Center for Brain Sciences, The Hebrew University of Jerusalem, Jerusalem 91904, Israel

## 

**Figure 1:**
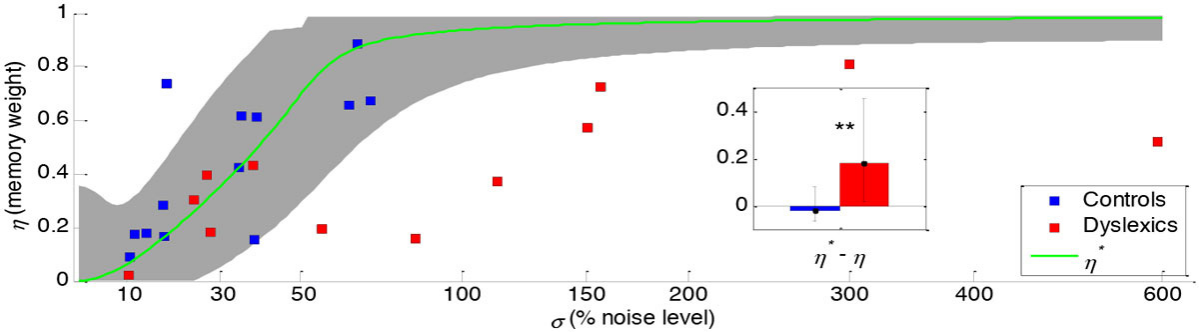
**Estimated parameters of the Implicit Memory Model**. Estimated values of *η *(weighting of implicit memory) as a function of estimated values of *σ *(percentage of internal noise) of Controls (blue) and Dyslexics (red). The optimal weighting η* is plotted in green. Gray area depicts the confidence interval of 2.5% below the best performance. **Inset**. Median deviation from optimal weighting of previous trials. Dyslexics' deviation is larger than Controls' (Mann-Whitney test, *Z *= 2.5, *P *< 0.01). Error bars denote inter-quartile range.

**Figure 2:**
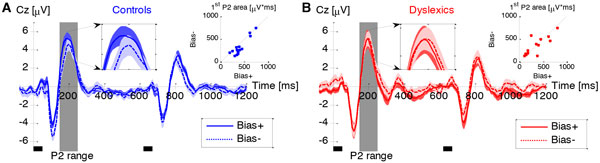
**Grand Average ERPs to the two-tone stimulation**. **A**. Controls **B**. Dyslexics. Trials are sorted according to the trial type, *Bias+ *(where the impact of previous trials improves performance) and *Bias- *(where the impact of previous trials impairs performance). Controls' P2 after the first tone differs between the two trial types. Dyslexics' evoked responses did not differ between the two trial types. Filled areas denote cross-subject SEM. Small black rectangles under the plots denote the temporal location of the two tones in the trial. **Middle insets**. P2 region enlarged; **Top right insets**. Single subject P2 area in *Bias- *versus *Bias+ *trials. The difference between the trial types is significantly larger among Controls than among Dyslexics (Condition × Group interaction: Mann-Whitney test, *z *= 2.5, *P *< 0.05).

Dyslexics are diagnosed for their poor reading skills. Yet, they characteristically also suffer from poor verbal memory, and often from poor auditory skills. We now hypothesize that dyslexia can be understood computationally as a deficit in integrating prior information with noisy observations. To test this hypothesis we analyzed performance in two tones pitch discrimination task using a two-parameter computational model. One parameter captures the internal noise in representing the current event and the other captures the impact of recently acquired prior information [1]. We found that dyslexics' perceptual deficit can be accounted for by inadequate adjustment of these components: low weighting of their implicit memory in relation to their internal noise (Figure 1). Using ERP measurements we found evidence for dyslexics' deficient automatic integration of experiment's statistics (Figure 2). Taken together, these results suggest that dyslexia can be understood as a well-defined computational deficit.
